# The association of the paraoxonase 1 Q192R polymorphism with coronary artery disease (CAD) and cardiometabolic risk factors in Iranian patients suspected of CAD

**DOI:** 10.3389/fcvm.2022.1037940

**Published:** 2023-01-09

**Authors:** Mina Darand, Amin Salehi-Abargouei, Mohammad Yahya Vahidi Mehrjardi, Awat Feizi, Seyed Mustafa Seyedhossaini, Gholamreza Askari

**Affiliations:** ^1^Department of Community Nutrition, School of Nutrition and Food Sciences, Nutrition and Food Security Research Center, Isfahan University of Medical Sciences, Isfahan, Iran; ^2^Nutrition and Food Security Research Center, Shahid Sadoughi University of Medical Sciences, Yazd, Iran; ^3^Department of Nutrition, School of Public Health, Shahid Sadoughi University of Medical Sciences, Yazd, Iran; ^4^Yazd Cardiovascular Research Center, Non-Communicable Disease Research Institute, Shahid Sadoughi University of Medical Sciences, Yazd, Iran; ^5^School of Public Health, Research Center for Food Hygiene and Safety, Shahid Sadoughi University of Medical Sciences, Yazd, Iran; ^6^Department of Biostatistics and Epidemiology, School of Health, Isfahan University of Medical Sciences, Isfahan, Iran

**Keywords:** PON1, Q192R polymorphism, rs662, lipid profile, coronary artery disease

## Abstract

**Introduction:**

The present study aimed to investigate the association of the paraoxonase 1 (PON1) Q192R polymorphism with coronary artery disease (CAD) and cardiometabolic risk factors in Iranian patients suspected of CAD.

**Methods:**

This cross-sectional study was conducted on 428 patients undergoing angiography. The data related to demographic information and physical activity were collected by valid and reliable questionnaires. The PON-1 genotypes were detected by the polymerase chain reaction-restriction fragment length polymorphism (RFLP-PCR) technique. The Gensini and SYNTAX score, anthropometric measurements, and biochemical and clinical parameters were measured by standard protocols.

**Results and discussion:**

Findings indicated that the odds of obesity was significantly higher in people with the RR genotype compared to the QQ genotype carriers (OR: 2.95 CI: 1.25–6.93, *P* = 0.014) and also odds of low high-density lipoprotein cholesterol (HDL-C) was marginally higher (OR: 2.31 CI: 0.97–5.49, *P* = 0.056). There was no significant association between other CAD risk factors with PON1 Q192R polymorphism (*P* > 0.05). Further analysis showed a significant interaction between sex and 192QR (*P* = 0.019) and 192 RR (*P* = 0.007) genotypes on body mass index (BMI). More specifically, the risk of obesity in men carrying the RR genotype was 3.38 times (OR: 3.38 CI: 1.08–10.58, *P* = 0.036). Also, a significant joint effect of the RR genotype and sex on HDL-C was seen (*P* = 0.003). The stratification based on sex showed that the risk of low HDL-C is significantly higher in women carrying the RR genotype (OR: 6.18 CI: 1.21–31.46, *P* = 0.028). A marginal sex-genotype interaction was also found in the risk of elevated alanine aminotransferase (ALT) (*P* = 0.057). In summary, the findings showed that the risk of obesity and low HDL-C was higher in people carrying the RR genotype. On the other hand, a Q192R polymorphism-sex interaction was observed on the risk of obesity, elevated ALT, and low HDL-C.

## Introduction

Coronary artery disease (CAD) is the leading cause of death and disability worldwide, accounting for approximately one-third of all deaths in people over age 35 (1). The disease causes about 7 million deaths and 129 million disabilities annually and imposes a substantial economic burden (2). Two categories of modifiable and non-modifiable risk factors have been identified in the incidence of CAD, of which atherosclerosis and dyslipidemia are the most important (3). On the other hand, twin studies have shown that the heritability of CAD ranges from 41 to 77 percent (4). The interpersonal differences observed in plasma biochemical factors and the risk of CAD in people with the same diet may be due to genetic differences (5). Due to the importance of genetic factors in CAD, many studies have investigated single nucleotide polymorphisms (SNP) of genes involved in the severity and occurrence of the disease (6). SNPs are genetic factors that exert the effect of genetic differences on food metabolism (7). The paraoxonase 1 (PON1) gene is located on the long arm of chromosome 7 (q21.3–22.1) and contains 26,857 bp. This gene includes nine exons and eight introns (8) and is highly polymorphic (9). The association of its common polymorphisms with lipid profile and susceptibility to CAD has been extensively investigated (10, 11). Numerous studies have shown that PON1 Q192R polymorphism (rs662) is associated with susceptibility to CAD (12, 13). For example, a meta-analysis study in 2019 showed that PON1 Q192R polymorphism increases the risk of CAD in people with type 2 diabetes, especially in Asian and Caucasian populations (14). A number of the studies reported that the RR genotype reduces high-density lipoprotein cholesterol (HDL-C)’s capacity to prevent low-density lipoprotein cholesterol (LDL-C) oxidation, so the 192R allele carriers have a higher risk of CAD than 192Q allele carriers (11). Other studies’ findings do not support these results. Conversely, another meta-analysis showed that the risk of CAD development is significantly higher in Q allele carriers (10).

Although many studies assessed the association between PON1Q192R polymorphism and the risk of CAD in the European populations, as far as we know, no study has investigated the association between CAD (based on Gensini and SYNTAX score) and the mentioned polymorphism in the Iranian ethnicity. The present study aimed to examine the association of the PON1 Q192R polymorphism with CAD and cardiometabolic risk factors in Iranian patients suspected of CAD.

## Materials and methods

### Participants

The present cross-sectional study was approved by the ethics committee of Isfahan University of Medical Sciences (Ethical approval code: IR.MUI.RESEARCH.1400.200), Iran, and was part of a larger research that its protocol was approved by the ethics committee of Shahid Sadoughi University of Medical Sciences, Yazd, Iran. Among the patients admitted under diagnostic coronary angiography in Afshar Hospital in Yazd, based on inclusion and exclusion criteria, 463 patients were enrolled. Patients aged 25–75 years with CAD who were willing and able to participate in the study were included. Patients with the following criteria were not included in the study: (1) history of cancer, heart failure, heart attack, percutaneous coronary intervention (PCI), coronary artery bypass grafting (CABG), chronic kidney disease stage 3 and above, specific liver disease or receiving medication for liver disorders, immunodeficiency, AIDS; (2) people with severe obesity [body mass index (BMI) above 40]; (3) pregnant and lactating women; (4) people who for any reason have limited food intake by mouth; (5) have a special diet. Non-response to many food frequency questionnaire items and not detecting the type of genotype led to the patient’s exclusion from the study. Finally, data from 428 patients were analyzed. Eligible individuals filled out written informed consent. The study was conducted in accordance with the Declaration of Helsinki.

### Assessment of the CAD

The extent and severity of CAD were assessed by Gensini and SYNTAX scores. For this purpose, the coronary angiogram was interpreted by an experienced cardiologist blinded for demographic and clinical data except for age and sex. Gensini and SYNTAX scores were calculated randomly for several participants by the second cardiologist. The Gensini score calculation begins by assigning a severity score to each identified coronary stenosis as follows: 1 point for ≤25% narrowing, 2 points for 26–50% narrowing, 4 points for 51–75% narrowing, 8 points for 76–90% narrowing, 16 points for 91–99% narrowing, and 32 points for total occlusion (100%). After that, each lesion score is multiplied by a factor that takes into account the importance of the coronary arteries and the lesion’s position in the coronary circulation (5 for the left main coronary artery, 2.5 for the proximal segment of the left anterior descending coronary artery, 2.5 for the proximal segment of the circumflex artery, 1.5 for the mid-segment of the left anterior descending coronary artery, 1.0 for the right coronary artery, the distal segment of the left anterior descending coronary artery, the posterolateral artery, and the obtuse marginal artery, and 0.5 for other segments). Finally, the Gensini score was obtained by summating the coronary segment scores. A higher Gensini score indicates a more intensive disease (15–17). The participants were categorized into two groups based on Gensini score: low Gensini score (<20) and intermediate-high Gensini score (≥20) (18).

The SYNTAX score was calculated through the internet-based SYNTAX calculator version 2.0.^1^ SYNTAX score algorithm comprising consecutive and interactional self-guided questions focusing on functional and anatomical parameters of the lesions with ≥50% stenosis in arteries with a diameter of ≥1.5 mm. The final SYNTAX score was obtained by summation of all lesion scores. The participants were categorized into two groups based on SYNTAX score: low SYNTAX score (<23) and intermediate-high SYNTAX score (≥23). A higher SYNTAX score indicates a more intensive disease (19, 20).

### Anthropometric and blood pressure measurements

In this study, a nutritionist measured weight using a portable digital scale and the body analyzer (Omron Inc., Osaka, Japan), with an accuracy of 0.1 kg, with minimal coverage and without shoes. Height was measured with an accuracy of 0.1 cm, using a wall-fixed measuring tape in a standing position with shoulders in normal alignment and no shoes. BMI was calculated as body weight (kg) divided by height squared (m^2^). Waist circumference (WC) was assessed by a flexible inelastic tape measure (i.e., the tape measure should not stretch when taking the measurement) in the standing position to the nearest 1 cm. The narrowest area between the iliac crest and the last rib was measured (21). We also recorded blood pressure measured by nurses before patients underwent angiography. BMI ≥ 30, WC > 102 cm for men and > 88 cm for women were considered obesity and abdominal obesity, respectively. The use of antihypertensive drugs or blood pressure ≥ 130 and/or ≥ 85 mm Hg was considered hypertension (22, 23).

### Biochemical assessment

Blood samples (4 ml) were obtained from all participants following overnight fasting. Two milliliters of blood samples were centrifuged at 2,500 rpm for 3 min to separate the serum from the blood cells. Buffy coats and remaining whole blood samples were stored at −80°C for DNA extraction and other biochemical tests. Triglyceride (TG), total cholesterol (TC), HDL-C, fasting blood sugar (FBS) (Biorex fars, Iran), alanine aminotransferase (ALT), and aspartate aminotransferase (AST) (Pars Azmun, Karaj, Iran) were measured by commercial kits. LDL-C concentration is also calculated using the Friedewald formula (24): LDL = TC − HDL − 1/5 (TG). Then, biochemical markers were categorized into normal TG (<150 mg/dL) or high TG (≥150 mg/dL), normal LDL-C (<130 mg/dL) or high LDL-C (≥130 mg/dL), normal HDL-C (≥40 mg/dL for men and ≥50 mg/dL for women) or low HDL (<40 mg/dL for men and <50 mg/dL for women) (22), normal TC (<200 mg/dL) or high TC (≥200 mg/dL) (25), normal ALT (<47 IU/L for men and <30 IU/L for women) or elevated ALT (>47 IU/L for men and >30 IU/L for women), normal AST (<30 IU/L) or elevated AST (>30 IU/L) (26), and normal FBS (<110 mg/dL) or high FBS (≥110 mg/dL) (22).

### DNA extraction and genotyping

**FIGURE 1:**
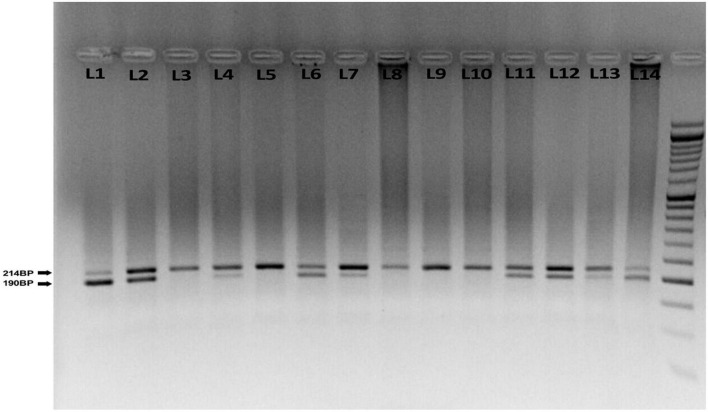
Agarose gel electrophoresis for the rs662 polymorphism of paraoxonase 1 (PONl) gene. The 214 bp bands correspond to wild homozygous QQ genotype produced one fragment, while 190, 214, and 24 bp correspond to heterozygous QR that produced three fragments. The 190 and 24 bp correspond to variant homozygous RR genotype produced two fragments. The 24 bp was invisible in the gel due to its fast migration speed. About 50 bp ladder marker (L1), QQ genotype (L3, 5, 8, 9, 10), QR genotype (L2, 4, 6, 7, 11, 12, 13), and RR genotype (L1, 14).

DNA samples were isolated from the white blood cell genome of the complete blood sample of the participants using the SimBiolab Blood Kit, according to the manufacturer’s protocol. The Q192R polymorphism (major allele: Q, minor allele: R), a fragment of 520 base pairs (bp) in exon 6 of the PON1 gene, was genotyped by the polymerase chain reaction-restriction fragment length polymorphism (PCR-RFLP) method. The PCR mixture was provided in a total volume of 20 μl containing 2 μl of genomic DNA, 10 μl of Master Mix (Amplicon, Denmark), 6 μl of water and 1 μl (10 pmol) of each oligonucleotide primer. Forward and reverse primer consists of AAACCCAAATACATCTCCCAGAAT and GCTCCATCCCACATCTTGATTTTA, respectively. PCR is performed by repeating three steps. First, DNA templates were denatured at 95°C for 5 min; amplification consisted of 45 cycles at 95°C for 15 s, annealing at 60°C for 30 s, extension at 72°C for 30 s, with a final extension at 72°C for 5 min. Amplified DNA (10 ml) was digested with 5 U restriction enzyme *Hin*fI (Fermentase, Germany) at 37°C, overnight to detect two different alleles, the 214 bp (Q allele), 24 bp and 190 (R allele). All products were visualized by electrophoresis in 2% agarose gel (SinaClon, Iran) at 90 V for 2.5 h. Three DNA fragments show with different lengths: homozygous RR (2 bands: 190 and 24 bp), heterozygous QR (3 bands: 214, 190, and 24 bp), and homozygous QQ (1 band: 214 bp). The 24 bp was invisible in the gel due to its fast migration speed (Figure 1).

### Assessment of other variables

General demographic data including age, smoking status, the medication used, and medical history were collected using valid and reliable questionnaires. Physical activity was assessed using International Physical Activity Questionnaire (IPAQ). Physical activity level was calculated based on metabolic equivalent task minutes per week (27). Persian translation validation of IPAQ has previously been confirmed by Moghaddam et al. (28).

### Statistical analysis

Continuous and categorical variables were expressed as mean ± standard deviation (SD) and frequencies (percentages). The chi-squared and one-way ANOVA tests were used to compare basic qualitative and quantitative variables between three genotypes (QQ, QR, and RR), respectively. We categorized the Gensini score, SYNTAX score, and cardiometabolic risk factors into categorical variables with two categories based on valid cut-off values for each variable. We evaluated their linear association with PON1 genotypes by using one-way ANOVA. Also, binary logistic regression was used to investigate the association of the PON1 genotypes with the Gensini score, SYNTAX score, and cardiometabolic risk factors in crude and multivariable-adjusted models. In multivariable models, the potential confounding effects of physical activity, gender, age, smoking status, alcohol consumption, the medication used (antihypertension drugs, antidiabetic drugs, and antihyperlipidemic drugs), and BMI (for high BMI and WC all mentioned confounders were adjusted except BMI) were adjusted. The results of logistic regression were reported as odds ratio (OR) and 95% confidence interval for the OR. The possible interaction effect of sex and the PON1 Q192R polymorphism on the Gensini score, SYNTAX score, and CAD risk factors was evaluated through logistic regression. When there was a significant interaction, the stratified analysis was done by sex, and the association of the PON1 Q192R polymorphism with the Gensini score, SYNTAX score, and CAD risk factors was analyzed. Analyses were performed using SPSS software version 24 (IBM Corp., Armonk, NY, USA). *P*-values less than 0.5 were considered significant.

## Results

### General characteristics of study participants

Characteristics of study participants according to PON1 genotypes are demonstrated in Table 1. The mean age of the participants was 56.74 years. The prevalence of PON1 Q192R polymorphism genotypes was QQ (47.4%), QR (45.1%), and RR (7.5%), and overall, 70 and 30% for Q and R alleles, respectively. The genotype PON1 Q192R polymorphism was distributed according to Hardy-Weinberg equilibrium (*p* > 0.05). Age, physical activity, medication use, gender, smoking status, and alcohol consumption were not different among genotypes of Q192R polymorphism.

**TABLE 1:** Characteristics of study participants according to PON1 genotypes.

Variables	Type of genotype			
	**QQ (203)**	**QR (193)**	**RR (32)**	***P* ^a^**
Age (y)	57.20 ± 9.61^b^	56.08 ± 8.84	57.84 ± 11.06	0.397
Physical activity (MET-minutes/week)	38.33 ± 6,619	4,696 ± 8,304	4,284 ± 604	0.203
Gender, male, n (%)	124 (61.1)^b^	125 (65.5)	20 (62.5)	0.709
**Medication use, n (%)**				
Antihyperlipidemic drugs, yes n (%)	76 (37.4)	66 (34.2)	11 (34.4)	0.786
Antihypertension, yes (%)	89 (43.8)	107 (55.4)	14 (43.8)	0.989
Antidiabetic drugs, yes (%)	62 (30.5)	62 (32.1)	13 (40.6)	0.524
Smoking status, n (%)				0.655
Never smoker	139 (86.5)	118 (61.1)	21 (65.5)	
Current smoker	57 (28.1)	66 (34.2)	10 (31.3)	
Former smoker	7 (3.4)	9 (4.7)	1 (3.1)	
Alcohol consumption, n (%)				0.704
Never smoker	190 (94.5)	180 (93.8)	29 (93.5)	
Current smoker	7 (3.5)	6 (3.1)	2 (6.5)	
Former smoker	4 (2)	6 (3.1)	0 (0)	

Met, metabolic equivalent for task.

^a^Obtained from Chi-squared test and one-way ANOVA for categorical and continuous variables, respectively.

^b^Continuous and categorical data are presented as mean ± (SD) and frequency (percentage). *P* < 0.05 was considered as statistically significant.

### Distribution of CAD risk factors across PON1 Q192R genotypes

The distribution of anthropometric indices, biochemical parameters, and other CAD risk factors across PON1 gene variants (QQ, QR, and RR) is reported in Table 2. HDL-C was marginally lower in carriers of at least one copy of the minor allele of Q192R polymorphism than in non-carriers (*P* = 0.090). Patients with the QR genotype have marginally higher FBS levels than carriers of the QQ and RR genotypes (*p* = 0.051). There was no significant difference between PON1’s genotypes regarding anthropometric indices, Gensini and SYNTAX score, and other biochemical and clinical parameters.

**TABLE 2:** Anthropometric indices, biochemical parameters, and other CAD risk factors across PON1 gene variants.

Variables	Type of genotype			
	**QQ**	**QR**	**RR**	*P*-value*
BMI	27.38 ± 4.06	27.36 ± 4.54	28.60 ± 4.55	0.315
WC (cm)	99.88 ± 14.28	99.25 ± 11.03	101 ± 9.89	0.747
Gensini score	33.89 ± 42.60	34.37 ± 41.12	42.66 ± 51.78	0.562
SYNTAX score	10.24 ± 12.52	11.03 ± 13.89	11.35 ± 12.83	0.808
TG (mg/dl)	152.60 ± 81.77	155.20 ± 88.42	159.40 ± 99.57	0.905
TC (mg/dl)	198.58 ± 102.40	206.02 ± 124.78	197.29 ± 114.94	0.803
LDL (mg/dl)	98.01 ± 39.81	99.61 ± 44.61	91.30 ± 41.62	0.598
HDL (mg/dl)	50.12 ± 13.14	48.45 ± 11.10	45.35 ± 9.83	0.090
FBS (mg/dl)	128.42 ± 55.86	143.12 ± 74.75	123.11 ± 51.22	0.051
SBP (mm/hg)	128.86 ± 15.18	128.53 ± 13.55	128.39 ± 14.16	0.969
DBP (mm/hg)	80.32 ± 11.90	78.43 ± 10.81	78.77 ± 12.59	0.263
ALT (IU/L)	56.64 ± 108.42	64.76 ± 108.79	28.35 ± 13.61	0.316
AST (IU/L)	44.49 ± 40.09	44.60 ± 28.95	41.15 ± 20.40	0.907

BMI, body mass index; WC, waist Circumference; HDL-c, high density lipoprotein cholesterol; LDL-c, low density lipoprotein cholesterol; TC, total cholesterol; TG, triglyceride; FBS, fast blood sugar; SBP, systolic blood pressure; DBP, diastolic blood pressure; ALT, alanine aminotransferase; AST, aspartate aminotransferase. Values are reported as mean ± (SD).

*Obtained from one-way ANOVA. *P* < 0.05 were considered as statistically significant.

### Association between PON1 Q192R genotypes and CAD risk factors

Crude and multivariable-adjusted odds ratios of the associations between PON1 Q192R polymorphism and Gensini and SYNTAX score and biochemical and clinical parameters are presented in Table 3. The crude logistic regression model identified that the odds of obesity was significantly higher in people with the RR genotype compared to the QQ genotype carriers (OR: 2.41 CI: 1.09–5.31, *P* = 0.029). This association remained unchanged after adjusting for age, gender, physical activity, medication use, alcohol consumption, and smoking status confounders (OR: 2.95 CI: 1.25–6.98, *P* = 0.014). Furthermore, homozygote people for the minor allele (RR genotype) compared to people with the QQ genotype have marginally higher odds of low HDL-C levels in the crude model (OR: 2.08 CI: 0.96–4.50, *P* = 0.061) and after adjustment of potential confounders (OR: 2.31 CI: 0.97–5.49, *P* = 0.056). In addition, the logistic regression model indicated that in patients with the RR genotype, the odds of elevated ALT were marginally lower than in people with the QQ genotype (OR: 0.29 CI: 0.07–1.09, *P* = 0.067). There was no significant association between other CAD risk factors with PON1 Q192R polymorphism (*P* > 0.05).

**TABLE 3:** Crude and multivariable-adjusted ORs (and 95% CIs) for the association of PON1 rs662 genotypes with anthropometric indices, biochemical parameters, and other CAD risk factors.

		Type of genotype			
	**QQ^£^**	**QR**	***P*-value^†^**	**RR**	***P*-value^†^**
**High BMI**					
Crude	1.00	1.17 (0.73–1.88)	0.511	2.41 (1.09–5.31)	0.029
Model 1	1.00	1.39 (0.83–2.32)	0.200	**2.95 (1.25**–**6.98)**	**0.014**
**High WC**					
Crude	1.00	0.81 (0.53–1.24)	0.351	0.89 (0.40–1.96)	0.782
Model 1	1.00	0.85 (0.53–1.38)	0.529	0.90 (0.37–2.19)	0.826
**High Gensini score**					
Crude	1.00	1.13 (0.57–1.69)	0.549	1.67 (0.77–3.60)	0.190
Model 1	1.00	1.06 (0.67–1.69)	0.777	1.25 (0.64–3.61)	0.338
**High SYNTAX sore**					
Crude	1.00	0.96 (0.57–1.63)	0.902	1.30 (0.52–3.27)	0.566
Model 1	1.00	0.96 (0.54–1.71)	0.911	1.23 (0.45–3.35)	0.675
**High TG**					
Crude	1.00	1.03 (0.67–1.58)	0.873	0.94 (0.42–2.08)	0.880
Model 1	1.00	0.91 (0.54–1.51)	0.725	0.84 (0.34–2.11)	0.722
**High TC**					
Crude	1.00	0.83 (0.54–1.28)	0.411	0.85 (0.38–1.89)	0.707
Model 1	1.00	0.85 (0.53–1.36)	0.506	0.91 (0.38–2.17)	0.833
**High LDL-C**					
Crude	1.00	1.29 (0.79–2.12)	0.304	0.75 (0.27–2.09)	0.588
Model 1	1.00	1.20 (0.70–2.07)	0.491	0.84 (0.29–2.45)	0.759
**Low HDL-C**					
Crude	1.00	0.92 (0.58–1.44)	0.724	2.08 (0.96–4.50)	0.061
Model 1	1.00	0.97 (0.58–1.61)	0.922	**2.31 (0.97**–**5.49)**	**0.056**
**High FBS**					
Crude	1.00	1.33 (0.88–1.99)	0.167	0.92 (0.43–1.97)	0.842
Model 1	1.00	1.44 (0.92–2.24)	0.105	0.90 (0.40–2.02)	0.801
**High SBP**					
Crude	1.00	1.33 (0.88–2.03)	0.172	1.19 (0.54–2.63)	0.655
Model 1	1.00	1.16 (0.73–1.84)	0.510	1.03 (0.44–2.40)	0.938
**High DBP**					
Crude	1.00	0.75 (0.45–1.26)	0.288	1.05 (0.42–2.62)	0.903
Model 1	1.00	0.70 (0.41–1.21)	0.207	1.01 (0.39–2.58)	0.979
**Elevated ALT**					
Crude	1.00	0.84 (0.51–1.39)	0.510	0.28 (0.08–1.01)	0.052
Model 1	1.00	0.82 (0.48–1.39)	0.465	**0.29 (0.07**–**1.09)**	**0.067**
**Elevated AST**					
Crude	1.00	1.63 (1–2.63)	0.046	0.93 (0.38–2.29)	0.887
Model 1	1.00	1.56 (0.94–2.61)	0.084	1.07 (0.41–2.78)	0.887

BMI, body mass index; WC, waist circumference; HDL-c, high density lipoprotein cholesterol; LDL-c, low density lipoprotein cholesterol; TC, total cholesterol; TG, triglyceride; FBS, fast blood sugar; SBP, systolic blood pressure; DBP, diastolic blood pressure; ALT, alanine aminotransferase; AST, aspartate aminotransferase.

^£^Reference group. Values are reported as odds ratio and 95% confidence interval. Model 1: adjusted for physical activity, gender, age, smoking status, medication used (antihypertension drugs, antidiabetic drugs, and antihyperlipidemic drugs), alcohol consumption, and BMI (for high BMI and WC all mentioned confounders were adjusted except BMI). P < 0.05 were considered as statistically significant.

^†^Obtained from logistic regression.

Bolded values are statistically or marginally significant.

### Interactions of sex and PON1 genotypes on BMI, HDL-C, and ALT

There was a significant interaction between sex and 192QR (*P* = 0.019) and 192RR (*P* = 0.007) genotypes in the association with BMI. We also identified an interaction between 192 RR and sex in the associations with serum levels of HDL-C (*P* = 0.003). A marginal sex-genotype interaction was also found in the risk of elevated ALT (*P* = 0.057). Accordingly, we conducted stratified analysis by sex and then evaluated the association of three PON1 genotypes with BMI, HDL-C, and ALT separately in men and women (Table 4).

**TABLE 4:** Multivariable adjusted odds ratios and 95% CI of rs662 polymorphism on BMI, HDL-C, and ALT stratified by sex.

Variables	Type of genotype				
	**QQ**	**QR**	***P* ^†^**	**RR**	*P* ^†^ *
**High BMI**					
Men	1	1.24 (0.58–2.63)	0.574	3.38 (1.08–10.58)	**0.036**
Women	1	1.71 (0.82–3.55)	0.147	2.47 (0.67–9.05)	0.171
Q192R* sex	1	1.45 (1.06–1.98)	0.019	2.16 (1.23–3.78)	**0.007**
**Low HDL-C**					
Men	1	0.89 (0.44–1.80)	0.765	1.10 (0.32–3.77)	0.873
Women	1	1.09 (0.50–2.36)	0.820	6.18 (1.21–31.46)	**0.028**
Q192R* sex	1	1.25 (0.91–1.72)	0.156	2.35 (1.32–4.18)	**0.003**
**Elevated ALT**					
Men	1	0.91 (0.45–1.84)	0.793	0.45 (0.08–2.46)	0.360
Women	1	0.56 (0.23–1.35)	0.202	0.20 (0.02–1.78)	0.150
Q192R* sex	1	0.84 (0.59–1.20)	0.353	0.41 (0.16–1.02)	0.057

Values are reported as odds ratio and 95% confidence interval.

^†^Obtained from logistic regression.

*Model is adjusted for physical activity, age, smoking status, medication used (antihypertension drugs, antidiabetic drugs, and antihyperlipidemic drugs), alcohol consumption, and BMI (for high BMI, all mentioned confounders were adjusted except BMI).

Bolded values are statistically or marginally significant.

### Gender-specific associations of genotypes and CAD risk factors

Stratified analysis based on sex indicated that only in the men population, RR genotype carriers had a higher risk of obesity than QQ genotype carriers (OR: 3.38, CI: 1.08–10.58, *P* = 0.036), while in women, the odds of obesity was not significantly different among the three genotypes. Classification based on sex also showed that the increased risk of low HDL-C in RR genotype carriers compared to QQ carriers is seen only in the women population and not in males (OR: 6.18, CI: 1.21–31.46, *P* = 0.028). We did not find a significant association between the ALT and any of the PON1 genotypes in men and women (Table 4).

## Discussion

Although many studies have assessed the association between PON1Q192R polymorphism and CAD in the European populations, as far as we know, no study has investigated the association between CAD (based on Gensini and SYNTAX score) and the mentioned polymorphism in the Iranian ethnicity, so the present study aimed to examine the association of the PON1 Q192R polymorphism with CAD and cardiometabolic risk factors in Iranian patients suspected of CAD. Although our findings generally indicated that the risk of obesity in RR genotype carriers was higher than in QQ genotype carriers, the stratification of patients based on sex showed that this association exists only in the men population. Limited studies have investigated the association of PON1 Q192R polymorphism with BMI as a risk factor related to CAD in general (29–32). In line with our results, Hassan et al. reported that BMI was significantly different among the three genotypes of PON1, and the RR genotype was associated with elevated BMI compared to the QQ genotype in CAD patients (29). Veiga et al. showed that the R allele frequency was significantly higher in obese women and associated with an increased risk of obesity (30). Similarly, Alharbi et al. suggested that BMI in diabetic patients carrying the RR genotype is significantly higher than the QQ genotype carriers (31). Contrary to the mentioned findings, another study showed that in patients with familial hypercholesterolemia, the QQ genotype was significantly associated with higher BMI than the RR genotype (32). One of the reasons for these discrepancies can be attributed to the interaction of genetic and environmental factors, including sex and race.

The results of the present study also illustrated that the risk of low HDL-C was marginally higher in the RR genotype carriers compared to the QQ genotype carriers. Although stratifying patients according to sex showed that the RR genotype is associated with a higher risk of low HDL-C only in women. Regarding other lipid factors (TG, TC, and LDL-C), no significant difference was seen between the three genotypes of PON1 Q192R polymorphism (QQ, QR, and RR). Wamique et al. reported that diabetic patients with the RR genotype have higher LDL-C and lower HDL-C levels vs. non-carriers (36). Similarly, in Hassan et al.’s study, RR genotype carriers had significantly lower HDL-C levels and higher TC, LDL-C, and TG levels than the QQ carriers (33). Interestingly, in another study, the R vs. Q allele was associated with increased HDL-C in white adults, whereas the opposite was true in blacks. Neither the Q nor the R allele was associated with LDL-C and TG in both races (34). On the other hand, in several studies, there was no significant difference regarding HDL-C across different Q192R genotypes in CAD patients (35–37). Among the reasons for the discrepancies in the findings of the studies, we can mention the interaction of genetic and environmental factors such as race, diet, and gender. The present study findings did not reveal an association between the risk of CAD (based on Gensini and SYNTAX score) and PON1Q192R polymorphism. Few studies have investigated the association of PON1 Q192R polymorphism with the risk of CAD (based on Gensini and SYNTAX scores). Similar to our research, Gu et al. reported no significant association between the Gensini score and Q192R polymorphism (38). Bayrak et al.’s study also showed Gensini scores distribution between different genotypes of Q192R polymorphism was not significantly different (39). In addition, the result of the present study indicated that in patients with the RR genotype, the odds of elevated ALT were marginally lower than in people with the QQ genotype. Studies in this field are limited. We found only one study that showed that liver enzymes (AST and ALT) were not associated with Q192R polymorphism (40).

Although the exact mechanisms of these associations have not been thoroughly investigated, the Q192R polymorphism seems to affect the activity of the paraoxonase 1 (PON1) enzyme (41, 42). The paraoxonase-1 (PON1) enzyme is one of the proteins constituting HDL-C particles and is responsible for its antioxidant and anti-inflammatory properties (43). PON1 has two lactonase and 3-esterase activities, which prevent oxidative changes in lipoproteins (44). More precisely, PON1 hydrolysis thiolactone to homocysteine and detoxifies it. Thiolactone induces atherogenic damage to the endothelium (45). PON1 inhibits LDL-C oxidation and lipid peroxides accumulation in macrophages. Decreased oxidized-LDL uptake is probably mediated via PON1 interaction with the scavenger receptor class B type 1 (SR-BI) on the macrophage’s surface, which results in the pro-inflammatory response suppression of macrophages (46). PON1 also diminishes monocyte chemotaxis and adhesion to endothelial cells, thereby preventing endothelium damage and atherosclerosis (47). Also, the research shows that incubating PON1 with HDL leads to a decrease in the expression of intercellular adhesion molecule (ICAM)-1 on endothelial cells, which helps reduce the progression of inflammation in the endothelium. PON1 also protects against the pro-inflammatory effects of oxidized phospholipids and lipopolysaccharide. Furthermore, PON1 reduces cholesterol biosynthesis by macrophages and increases cholesterol efflux from LDL-C (48). Some research works, but not all (49), have shown that in the RR genotype carriers, PON1 enzyme activities decreases, which increases oxidative stress and inflammation, and all these events may be related to the incidence of atherosclerosis and CAD (50, 51).

The present study has strengths, which are briefly addressed. First, this study is the first study that measures the association between the risk of CAD (based on Gensini and SYNTAX score) with Q192R polymorphism in patients undergoing coronary angiography. Second, to obtain the association, confounding factors were adjusted. On the other hand, the present study has limitations that should not be ignored. Due to the cross-sectional nature of the study design, it is not possible to derive a causal association. This study was done only on patients in Iran, so it cannot be generalized to the whole world. Third, due to budget limitations, it was not possible to measure the PON1 of serum, so it is difficult to talk about possible mechanisms. Fourth, in this study, we could not detect some patients’ genotypes.

## Conclusion

The findings showed that the risk of obesity and low HDL-C was higher in people carrying the RR genotype. On the other hand, a Q192R polymorphism-sex interaction was observed on the risk of obesity, elevated ALT, and low HDL-C. Although a significant association between PON1 Q192R and the associated CAD risk factors was observed, more innovative observational and mechanism-based studies are needed to confirm this association and identify potential mechanisms.

## Data availability statement

The data can be found here (https://www.ncbi.nlm.nih.gov/snp/rs662).

## Ethics statement

This study was approved by the Institutional Review Board of Isfahan University of Medical Sciences (Ethical approval code: IR.MUI.RESEARCH.REC.1400.200). The patients/participants provided their written informed consent to participate in this study.

## Author contributions

GhA and AS-A contributed to the conception and design of the study. AF and MD performed the statistical analysis. MD wrote the first draft of the manuscript. MYVM and SMS contributed to the data collection. All authors contributed to manuscript revision, read, and approved the submitted version.

## Footnotes

1
http://syntaxscore.org/calculator/syntaxscore/frameset.htm
